# Perioperative outcome and predictors of success after tracheal resection

**DOI:** 10.1007/s00423-026-04175-3

**Published:** 2026-08-12

**Authors:** Laura V. Klotz, Birgit Khandanpour, Martin E. Eichhorn, Werner Schmidt, Markus Scheiner, Dennis Aliev, Feyza Özkan, Judith M. Brock, Konstantina Kontogianni, Hans Hoffmann, Felix J.F. Herth, Hauke Winter, Florian Eichhorn

**Affiliations:** 1 Department of Thoracic Surgery, Thoraxklinik, University Hospital Heidelberg, Röntgenstraße 1, Heidelberg, 69126 Germany; 2 Translational Lung Research Center Heidelberg (TLRC-H), Member of the German Center for Lung Research (DZL), Heidelberg, Germany; 3 Department of Anaesthesiology, Thoraxklinik, University Hospital Heidelberg, Röntgenstraße 1, Heidelberg, 69126 Germany; 4 Department of Pneumology, Thoraxklinik, University Hospital Heidelberg, Röntgenstraße 1, Heidelberg, 69126 Germany; 5 Department of Thoracic Surgery, Klinikum rechts der Isar, TU Munich, Ismaninger Straße 22, Munich, 81675 Germany

**Keywords:** Tracheal stenosis, Tracheal reconstruction, Thoracic surgery, Endoscopy

## Abstract

**Purpose:**

Tracheal stenosis (TS) leads to progressive ventilatory restriction and endoscopic interventions for symptomatic relief have often palliative character. Resection of the narrowed segment can result in sustainable long-term outcome. We analyzed our experience in tracheal reconstructive surgery with focus on perioperative management and procedural morbidity.

**Methods:**

We conducted a retrospective study of prospectively obtained clinical data from patients undergoing tracheal surgery at the Thoraxklinik Heidelberg between July 2000 and September 2023. Aim of the study was to identify clinical variables with impact on the perioperative course and individual outcome.

**Results:**

138 patients underwent tracheal surgery for TS in our department. Main indications were post-tracheostomy stenosis (*n* = 77, 55.8%), malignant tracheal tumor (*n* = 27, 19.6%) and idiopathic tracheal stenosis (*n* = 22, 15.9%). The latter was observed more frequently in females (95.2%, *p* < 0.001). Most patients were extubated in the surgical theatre (83.3%) or immediately in the early postoperative period (< 24 h, 8.7%). No 30-day or 90-day mortality was observed. 13 patients (9.4%) developed symptomatic tracheal restenosis. Only seven patients (5.1%) required endoscopic intervention. Postoperative prolonged ventilation (> 24 h postop) was associated with development of anastomotic insufficiency and wound infections. Female sex and history of idiopathic stenosis were significant risk factors for recurrent stenosis.

**Conclusion:**

Reconstructive surgery for TS results in favorable functional outcome in selected patients and is associated with a low morbidity and mortality. Patient selection and perioperative management requires an experienced multidisciplinary team.

## Introduction

Tracheal stenosis (TS) is a rare but potentially life-threatening condition characterized by segmental or circumferential restriction of the tracheal lumen. It is typically associated with progressive ventilatory distress and stridorous breathing. Common causes for development of TS are history of tracheostomy, (traumatic) intubation, and tracheal neoplasms. Idiopathic tracheal stenoses (ITS) are related to chronic inflammatory processes leading to (mostly subglottic) fibrotic tracheal narrowing [1–3]. Endoscopic treatments for TS include cryoablation, balloon dilation, incision with electric knife, mechanical debulking and stent placement. However, the long-term efficacy of these interventions can be inconsistent and subsequent surgical procedures are often necessary to obtain a persistent solution. This was impressively demonstrated in a large series by Wright and colleagues with a 96% success rate in 301 patients requiring tracheal resection and reconstruction for postintubation stenosis [4, 5]. Surgical management of TS varies along the etiology, severity, and location of the TS and perioperative risks must be balanced against complications associated with less invasive treatments [6]. The surgical gold standard for the treatment of severe TS is the circumferential resection of the restrictive segment with reconstruction by end-to-end anastomosis [7, 8].

Standardized surgical techniques and developments in anesthetic management have led to improved functional outcomes for patients with TS. The most significant complications necessitating a surgical intervention following tracheal resection were anastomotic insufficiency or tracheal restenosis with the need for redo-procedures. Studies have emphasized the importance of thorough preoperative evaluation, as repetitive endoscopic interventions for tracheal widening have been shown to negatively impact surgical outcomes due to postprocedural swelling and granulation tissue formation [5, 9]. Furthermore, the likelihood of postoperative complications may increase with obesity, failure of postoperative extubation and a prior tracheostomy [2]. Therefore, a multidisciplinary approach involving pneumologists, otolaryngologists, thoracic surgeons, and anesthesiologists is essential to ensure optimal outcomes. The objective of our study was to examine the experience with surgery for TS in our high-volume center, with focus on perioperative morbidity and surgical management.

## Patients and methods

### Patient selection and baseline assessment

Patients were selected retrospectively from a prospectively maintained database of individuals who underwent tracheal surgery at Thoraxklinik Heidelberg between July 2000 and September 2023. Patients with benign, inflammatory or malignant airway strictures requiring resection of any part of the trachea and/or main carina were analyzed. Patients with concomitant major pulmonary resections (e.g. sleeve pneumonectomy) were excluded from further analysis. The Heidelberg University Hospital Institutional Ethics Committee approved the collection and analysis of data from patients’ medical records, waiving the requirement for individual informed consent (S-174/2019).

Each patient was discussed by the multidisciplinary team comprising specialists in thoracic surgery, interventional pulmonology and anesthesiology. All patients underwent rigid bronchoscopy prior to surgery for detailed local evaluation of the stenosis. Endoscopic diagnostic (e.g., biopsy) or palliative interventions (e.g., reopening of stenosis by tracheal dilatation, incision or airway stenting) were applied individually. Each endoscopic workup included concise examination of the stenotic segment (location, length, distance to vocal cords). The diameter of the remaining airway lumen was graded according to the Myer-Cotton classification [10]:

- *Grade I: no obstruction to 50% obstruction;*
- *Grade II: 51% to 70% obstruction;*
- *Grade III: 71% to 99% obstruction;*
- *Grade IV: no detectable lumen.*

Patient’s demographics comprised information on gender, age, the underlying disease or mechanism causing TS, history and the number of preoperative interventions, intra- and postoperative course including procedural parameters (e.g. surgical access, length of resected segment, time to extubation, length of stay on intensive care unit) and complications (e.g. rate of recurrent stenosis, development of anastomotic leakage).

Idiopathic tracheal stenosis was defined as a stenosis of the subglottic airway and/or upper trachea in the absence of any identifiable underlying cause. Patients were classified as idiopathic if there was no history of prior airway instrumentation (e.g., endotracheal intubation or tracheostomy), external trauma, infection, systemic autoimmune or inflammatory disease, or malignancy. In line with established definitions of idiopathic subglottic stenosis, most cases were in the subglottic region; however, patients with extension into the upper trachea were also included, provided no causative factor could be identified.

### Surgical procedure

**Fig. 1:**
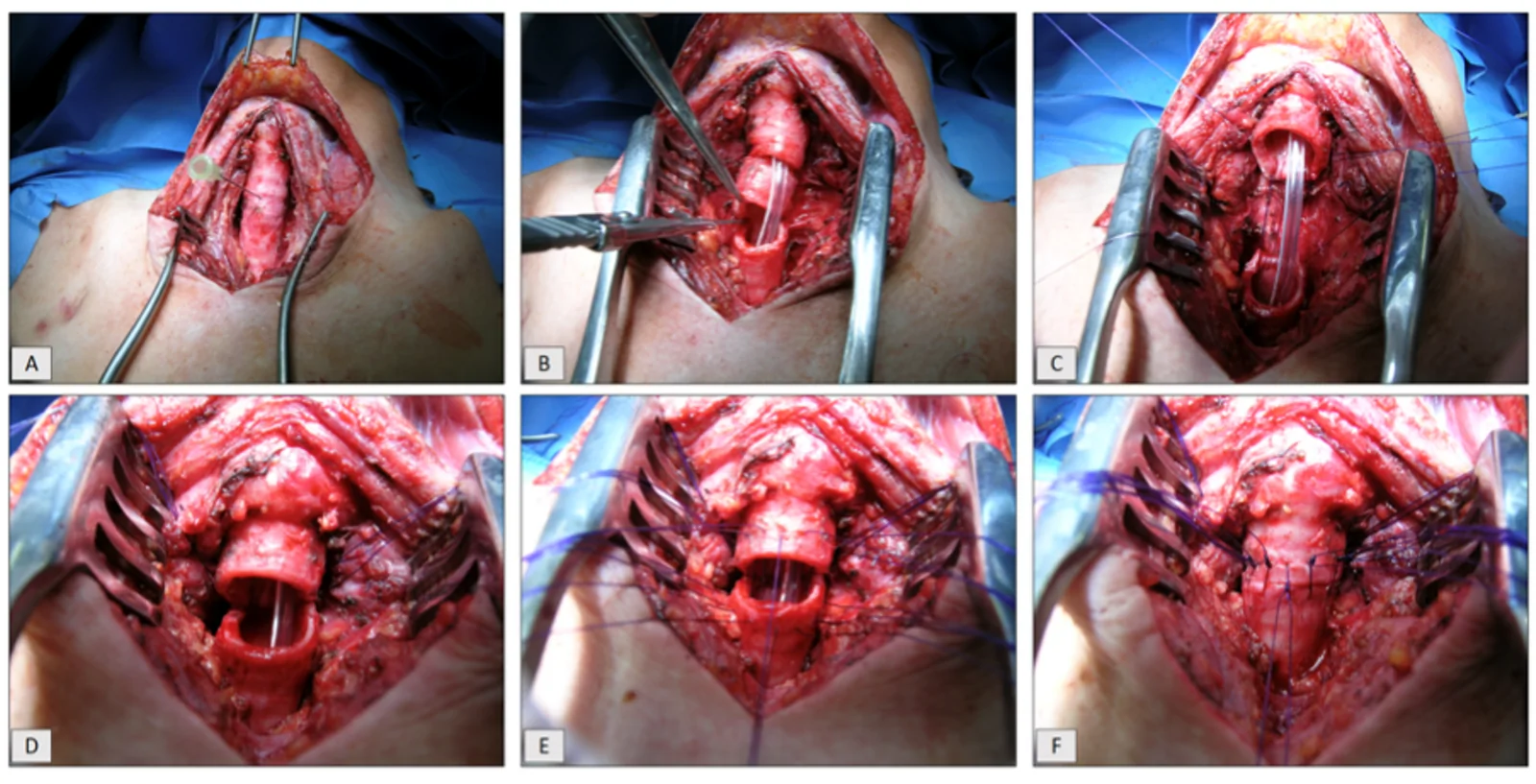
Essential procedural steps during resection of cervical TS. A: Location of the stenotic segment after exposure of the trachea. Endoscopic orientation via the anesthesiologist (not shown). B: Circumferential intercartilaginous incision above and below the stenosis, jet catheter introduced. C: Threads (3-0 PDS) laid out for the continuous seam of the rear wall (“parachute technique”, pars membranacea), bilateral corner threads (PDS 3-0) in place. D: completed continuous suture of the posterior tracheal wall. E: 6-8 pericartilagineous single-button sutures before adaption of the anterior wall of the trachea. F: Complete tracheal anastomosis

Access to the surgical field was planned based on pre- and intraoperative findings and location of the tracheal segment involved. All patients had balanced anesthesia with laryngeal mask for high subglottic stenosis or, for strictures within the mid or lower portion of the trachea, with endotracheal intubation at baseline. High frequency jet ventilation was applied during tracheal resection and reconstruction [11]. Cross-field ventilation was available on demand as a rescue maneuver in case of inadequate oxygenation. Flexible intraoperative bronchoscopy ensured sufficient formation of anastomosis. Patients were extubated in the operating theatre whenever possible. The institutional principles of tracheal resection and reconstruction followed the techniques described by Grillo et al. [12, 13]. The most relevant surgical junctions with respect to the location and the extent of the stenotic tracheal segment to be removed are described as follows:

Collar transversal incision above the sternal notch. Division of the platysma. Thyroid gland was cranialized or divided through dissection of the isthmus and the pretracheal fascia. The trachea is explored beginning from the ventral cartilaginous portion. Blunt paratracheal dissection and mediastinum enables adequate mobilization of the trachea (Fig. 1A). Partial craniocaudal sternal split facilitates access to a stenotic area located just above the middle third of the trachea. Intercartilaginal incision of the anterior tracheal wall cranially and caudally (Fig. 1B). In case of (partial) resection of the cricoid cartilage, the shape of the cranial incision determines the caudal resection margin to secure a latter precise re-adaption and anastomosis. Circumferential exposition of the trachea at the level of the stenosis by dissection between the posterior wall of the trachea and the esophagus; distal dissection beyond the resection margin should be avoided to preserve the vascular supply.

Resections of the middle and lower third of the trachea including the bifurcation demanded a transsternal approach or right-sided thoracotomy (for access to the lower third).

Tracheal anastomosis was performed using absorbable 3 − 0 or 4 − 0 PDS (polydioxanone suture). Running suture of the posterior tracheal using the “parachute” technique (Fig. 1 C, D). 6–8 pericartilagineous single-button sutures for anastomosis of the anterior wall (Fig. 1 E, F) [14]. In case of isolated carinal resection, a side-to-side suture of the main bronchi was implemented, followed by a running suture to readapt the distal part of the trachea and the double-barrel-shaped neo-carina. The tracheal anastomosis was wrapped with vital adjacent tissue depending on the location of the removed trachea segment (e.g. thyroid gland, strap muscles, pericardial fat pad).

**Fig. 2:**
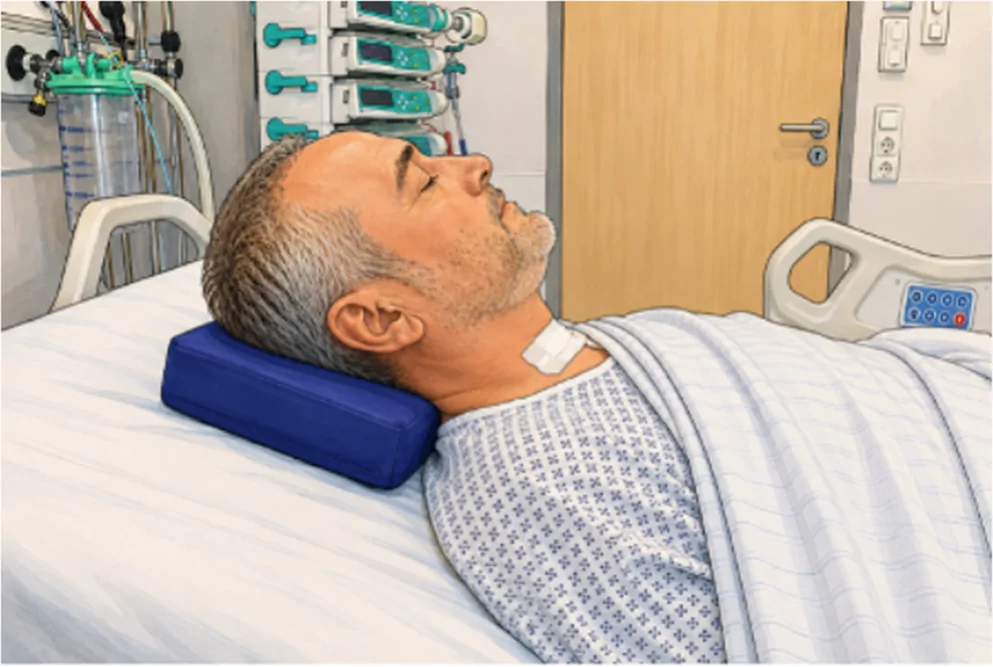
Illustration of the patient's postoperative positioning. The pillow support prevents unintentional dorsal flexion of the head (Created with ChatGPT)

All patients received a single-shot antibiosis (e.g. fluoroquinolone) to prevent infection. Prolonged or postoperative antimicrobial therapy was not routinely administered unless clinically indicated. Within the first days, the head of the patients was gently fixed with a soft shell-shaped head cradle to prohibit unintentional retraction of the neck preventing tension to the anastomosis (Fig. 2). A guardian chin-to-chest stich was used in sole early cases in this series.

### Follow up

All patients underwent at least one bronchoscopy postoperatively upfront to hospital discharge. Follow-up visits were scheduled individually on demand. Follow-up information was obtained from the institutional database if available. Surgery-related complications and those occurring within the first 30 and 90 postoperative days were analyzed. Superficial wound infections and insufficiencies of the anastomosis were defined as early complications whereas recurrent stenosis (either symptomatic or asymptomatic) was defined as late complication. Persistent functional vocal cord paralysis due to intraoperative injury of the recurrent laryngeal nerve was reported separately as well as resulting procedures (e.g. permanent tracheostomy). Patients without re-consultation or documented contact to our outpatient service were assumed to lack symptomatic recurrent stenosis. Recurrence free-survival (RFS) was defined as the time from discharge until the development of a symptomatic tracheal re-stenosis requiring any intervention.

### Statistical analysis

The Kaplan Meyer method was used to calculate RFS. Factors associated with development of perioperative complications or re-stenosis were assessed by testing with Pearson Chi Square test. A p-value of less than 0.05 was considered statistically significant.

## Results

**Fig. 3:**
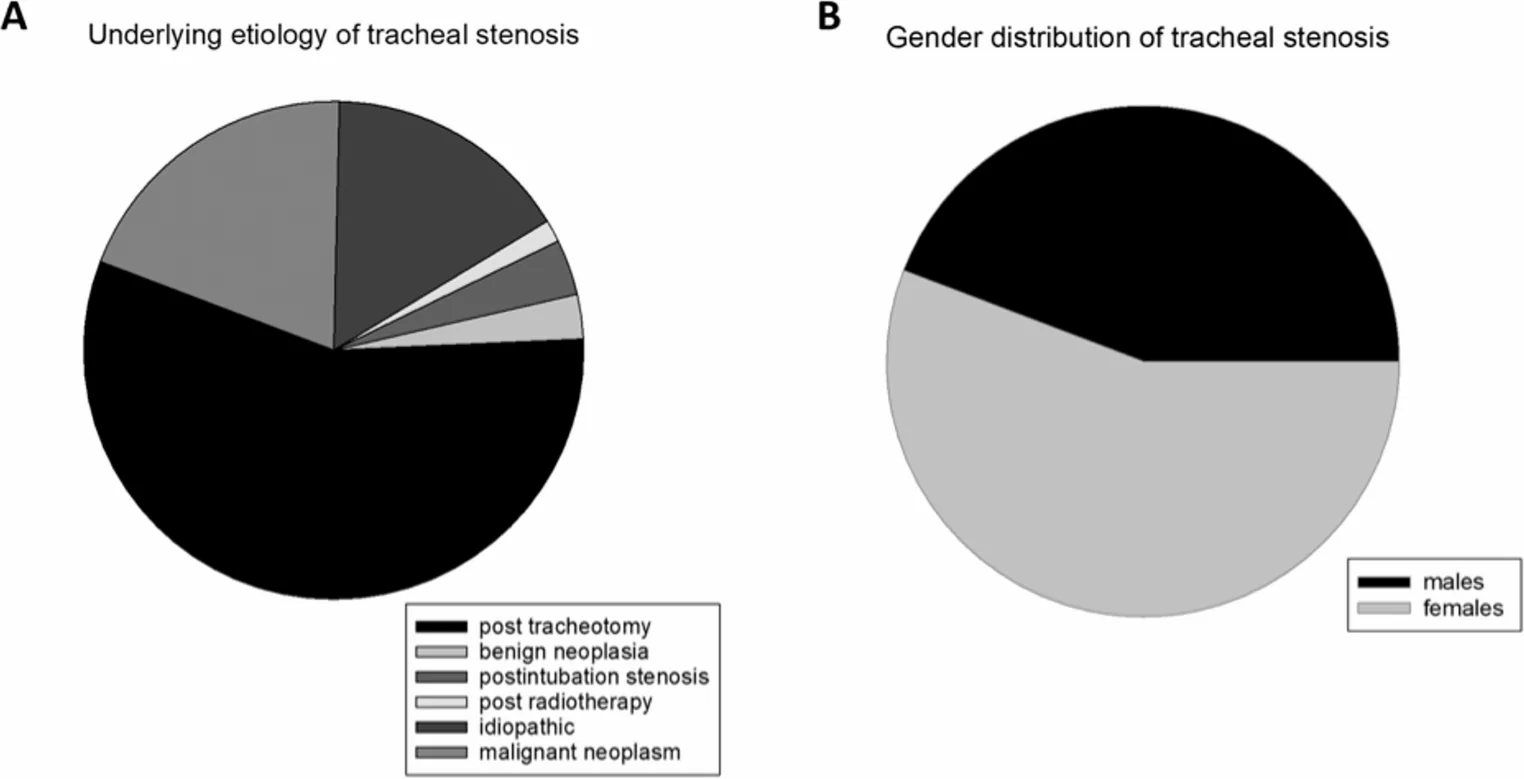
Distribution of relevant patient characteristics. **A** Patients were stratified by the underlying disease causing TS. There were 77 patients due to tracheotomy for prolonged ventilation, 27 patients with a malignant neoplasm and four with a benign neoplasm, 22 patients with idiopathic subglottic tracheal stenosis, two patients who had undergone radiotherapy and five patients who had a history of intubation (see also Table 1). **B** Gender distribution showed a higher proportion of female patients (n = 77; 55.8%) than male patients (n = 61; 44.2%)

138 patients (61 male, 44.2%) at a mean age of 56 years (± 17.5) underwent tracheal surgery between July 2000 and September 2023 at our high-volume thoracic surgery center. The majority of stenoses (55.8%) were associated with prolonged ventilation following tracheostomy. 20 of 21 idiopathic tracheal stenoses (95.2%) were found in female patients (*p* < 0.001; Fig. 3). 61 patients had at least one endoscopic intervention for symptomatic treatment of the stenotic area. Simple incisional widening (20 patients, 14.4%) alone or in combination with subsequent dilatation either by rigid scope or balloon (30 patients, 21.7%) were the most common procedures.11 patients (7.9%) had a history of stent implantation as an interventional treatment for their TS. Stent implantation prior to surgery was necessary in 11 patients (7.9%) (Table 1).

**Table 1:** Patient characteristics

	*n* (%) or median ± standard deviation
*n*	138 (100)
Gender	
male	61 (44.2)
female	77 (55.8)
Age at surgery [years]	56.2 ± 17.6
Cause of tracheal stenosis	
tracheotomy for prolonged ventilation	77 (55.8)
malignant tracheal tumor	27 (19.6)
idiopathic	22 (15.9)
post radiotherapy	2 (1.4)
post intubation	5 (3.6)
benign neoplasia	4 (2.9)
infectious	1 (0.7)
Comorbidities	
Cardiovascular	57 (41.3)
Diabetes	23 (16.7)
Obesity	12 (12.3)
Number of bronchoscopic interventions	
0	77 (55.8)
1	47 (34.1)
≥ 2	14 (10.1)
Preoperative bronchoscopic intervention	
none	77 (55.8)
ablation / incision only	20 (14.4)
dilatation	30 (21.7)
stent	11 (7.9)
Cotton-Myer classification	
grade 1	18 (13.0)
grade 2	64 (46.4)
grade 3	56 (40.6)
grade 4	0 (0)

Mean interval from first endoscopic diagnosis of the symptomatic TS to surgery was 8 months. Most pathologies were in the upper third of the trachea (121 patients, 87.7%), and involvement of the cricoid cartilage was observed in 11 patients (8%). Isolated resection of the carina without lung resection was performed in 5 patients (3.6%).

A collar surgical approach to the trachea was performed in 114 patients (82.6%). Sternotomy (12.3%) and right-side thoracotomy (5.1%) were chosen for resections involving the mid (6 patients, 4.3%) and lower parts of the trachea (6 patients, 4.3%). Uneventful postoperative extubation was possible in the majority of patients (127; 92%) (Table 2). However, prolonged ventilatory support (> 24 h) was necessary in 11 cases due to laryngeal edema as the predominant cause (8 patients). 

**Table 2:** Peri- and postoperative parameters

	*n* (%) or median ± standard deviation
*n*	138 (100)
Time to surgery (from first diagnosis), months, mean (malignant lesions excluded)	8.05 (0-270)
Surgical approach	
collar incision	114 (82.6)
thoracotomy	7 (5.1)
sternotomy	17 (12.3)
Resection type	
tracheal segmental resection	127 (92.0)
cricotracheal resection	11 (8.0)
Length of resected tracheal segment (cm, mean)	2.7 (0.9-7)
Prolonged postoperative ventilation	
no	115 (83.3)
less than 24 h	12 (8.7)
more than 24 h	11 (8.0)
Stay in the intensive care unit [days, median]	1.0 (0–29)
Deaths within 30/90 days	0/0
Hospital stay [days, median]	11.0 (4–69)
Mean/median follow-up (months)	23.2/5
Freedom of recurrent stenosis	82.1% at five years
Recurrent stenosis	
Asymptomatic	6 (4.3)
Symptomatic	7 (5.1)
Recurrent nerve palsy	4 (2.9)
Short-term complications	
Wound infection	6 (4.3)
Anastomotic leakage	3 (2.2)

### Postoperative Outcome

No deaths were observed within 30 and 90 days postoperatively. Superficial cervical wound infections occurred in 6 patients, and insufficiencies of the tracheal anastomosis were observed in 3 patients (Table 2). One patient was managed conservatively, two patients needed surgical reconstruction of the anastomosis. 13 patients (9.4%) experienced tracheal re-stenosis related to scarred stricture of the anastomotic region. Endoscopic interventions (incision, widening) were indicated in 7 symptomatic patients (5.1%). The remaining 6 patients (4.3%) were asymptomatic with diagnosis of only minimal endotracheal strictures during endoscopic follow-up.

**Fig. 4:**
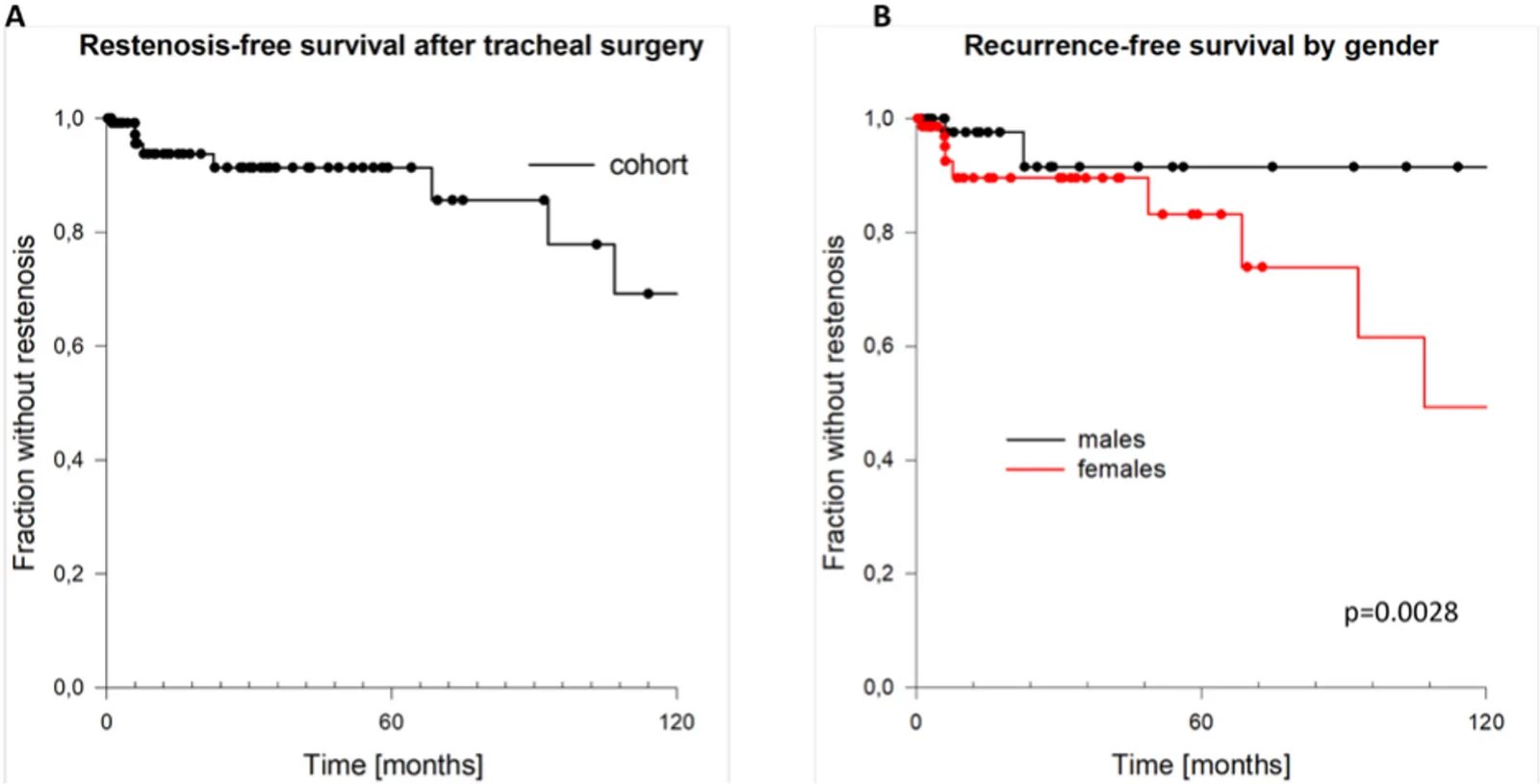
Recurrence-free survival of patients after tracheal resection. **A** The recurrence-free survival of patients after tracheal resection is shown 12 months after surgery. **B** The recurrence-free survival of patients after tracheal resection was distributed by gender, with a significantly higher incidence of restenosis being observed in female patients compared to males (*p* =0.028)

97.8% of patients were without symptomatic stenosis at 12 months (Fig. 4A). The development of early complications (wound infection, anastomotic insufficiency) was not associated with patient sex, age, comorbidities, previous tracheal surgery (e.g. tracheostomy), the number of preoperative endoscopic interventions or the length of the resected tracheal segment (> 4 cm). Early complications occurred more frequently in patients with prolonged postoperative intubation > 24 h; (27% vs. 5%; *p* = 0.034). Recurrent stenoses were found to be more prevalent in female patients (14.3% vs. 3.3%, *p* = 0.028; Fig. 4B) and in patients with idiopathic TS (31.8% vs. 5.2%; *p* < 0.001). Recurrent stenosis as a late complication was not associated with prolonged ventilation (*p* = 0.277) but patients with postoperative wound infections and anastomotic insufficiency tended to experience latter recurrent stenosis (30% vs. 7.8%, *p* = 0.054).

## Discussion

Tracheal resection and anastomosis can achieve an excellent functional outcome for patients suffering from multifactorial TS. We report our single institutional experience with 138 consecutive patients. No postoperative deaths were observed within the first 3 postoperative months, and only 5.1% of patients developed symptomatic re-stenosis. Short and long-term complications were more frequent in patients with prolonged postoperative ventilation, and female gender was associated with recurrent stenosis. Approximately 60% of our patients had TS related to prolonged intubation or post tracheostomy. Other high-volume centers report up to 75% of their stenoses caused by cuff-related tracheal strictures. However, the overall incidence has decreased over the years due to continuous technical advances [15]. Malignant stenoses are rare, as primary tracheal cancers represent only 0.2%-0.4% of neoplastic diseases [16]. The second most common benign cause of TS was idiopathic stenosis, representing 15% of our patients. We observed a significant predominance in female patients, which is consistent with findings in literature [17, 18].

The number or type of preoperative endoscopic interventions did not significantly affect the postoperative course. However, repetitive endoscopic treatment delayed the final surgical resection of the narrowed tracheal segment to a mean time of more than 9 months. Patel et al. report a median interval of 5 months between the first dilatation and formal resection [18]. Sahin et al. analyzed 40 patients that finally underwent tracheal surgery but needed a median of 3 preoperative endoscopic interventions for rigid dilatation [19].

Procedure-related mortality is less than 1% in most series and few observed deaths were associated to “non-airway” comorbidities [20, 21]. However, a compromitted healing of the trachea and the surrounding connective tissue may affect the integrity of the anastomosis and is a severe concern that requires a meticulous interdisciplinary management. The incidence of postoperative anastomotic complications ranges from 1 to 9% in the literature [22, 23]. We observed anastomotic insufficiencies in only three of our patients (2.2%), two of whom required surgical revision. No patient died post operatively. The probably largest series of tracheal resections by Wright et al. shows anastomotic complications of any severity (insufficiencies, granulation tissue, restenosis) in 81 of 901 patients (9%). Of note, anastomosis insufficiencies were observed in only 37 patients (4%) [21].

The most significant technical factor to assure adequate healing is to avoid tension to the anastomosis. Resections of tracheal segments of more than 4 cm length have been shown to be significantly associated with anastomotic insufficiencies in other series [21, 23]. If longer resections are necessary, intraoperative cervical or intrathoracic tracheal release maneuvers may facilitate tension free anastomosis [24]. We did not observe a higher incidence of anastomotic complications in patients with extended tracheal resection (> 4 cm) but found only 13 patients (9.4%) matching these criteria in our cohort.

Postoperative excessive hyperextension of the neck may additionally comprise healing of the new anastomosis. It depends greatly on the respective training in tracheal surgery whether surgeons may prevent unintended dorsal neck movement by introducing a suture from the patients chin to the ventral chest (“guardian chin stich”) or to supply patients with an individualized head cradle instead [25, 26]. Kaiser and Cooper, both with immense experience in tracheal surgery, commented in a controversial discussion of both tools correctly, that the most important technical issue is a carefully fitted creation of the anastomosis preserving a maximum of blood supply [25]. The major criticism of a (incorrectly too tight) chin-stich causing forced postoperative neck flexion is that it may pathologically stress the spinal cord resulting in severe myelopathies (paraplegia, hemiplegia) rarely observed after tracheal surgery [26, 27]. Our department abandoned that technique since we resected a TS in a 20-year-old female with complicated postoperative course. Protective intubation and a chin-stich were introduced following the final tracheal revision to relieve tension on the anastomosis. Indeed, severe tetra-paresis was clinically apparent after successful weaning. Further spine imaging revealed a previously unknown coexisting tethered-cord syndrome with fixed caudal spinal cord unable to longitudinally compensate cervical flexion [27, 28].

Cervical wound infections may occur within the postoperative period with an incidence of around 4% [8, 29] as low as observed in our patient cohort (6 patients; 4.3%). Obviously, these patients require the unattached attention of the surgical team to intervene as early as necessary to avoid any infection of the tracheal suture line. Clinical observation and antibiotic treatment often leads to successful results [20]. Preexisting diabetic metabolic state has been identified by Wright et al. as an individual risk factor for the development of complications [21]. Of note, more recent data from multicenter observational trials including 267 patients could confirm neither diabetes nor obesity alone as independent risk factors, but reported that systemic comorbidities (lung or cardiac insufficiency, abdominal disorders and metabolic disorders) were associated with a higher rate of complications following tracheal reconstruction [30]. In our series, preexisting cardiovascular or immunosuppressive comorbidities did not lead to higher morbidity. However, we identified prolonged mechanical ventilation for more than 24 h after surgery as a significant prognostic factor for the development of early complications (wound infection, anastomotic leakage). Laryngeal edema was causally found in patients without restoration of adequate spontaneous breathing. Excessive endotracheal swelling was then followed by impaired local microcirculation and ischemia to the surgical field [31]. Therefore, extubation and spontaneous breathing within the operation room or immediately within few hours after surgery is strongly advised to enable an uneventful recovery of the anastomosis and paratracheal soft tissue^[22]^.

Patients with surgical site infections and/or insufficience of tracheal anastomosis tended to develop recurrent TS. Delayed wound healing with endotracheal tissue overgrowth and formation of granulation tissue has been described as a common complication after tracheal resection or tracheostomy [32–34]. Notably, the rate of overall symptomatic restenosis needing redo-procedures was only 5% in our series and is comparably low (ranging from 0%-20%) [23, 32, 35, 36]. We observed that female patients and related thereto patients with idiopathic subglottic stenosis experienced a higher risk of postoperative airway strictures. In idiopathic subglottic stenosis, persistent cytokine-derived mucosal inflammation results in an accumulation of fibrotic tissue and enables formation of endotracheal scar. In fact, these local changes in tissue integrity can also promote the development of recurrent stenosis after tracheal surgery [37]. Given the higher incidence of redo-procedures in female patients in our series, a hormonal influence on the development TS can be considered [38]. Notably, Fiz et al. demonstrated differences in estrogen and progesterone-receptor expression on tissue-resident fibroblasts when comparing idiopathic to non-idiopathic tracheal specimen. The authors assumed that gender-related hormonal imbalances may foster inappropriate inflammation and increase stenosis susceptibility [39].

The main limitations of our study are the retrospective design and the lack of continuous long term endoscopic follow-up. However, recent work showed that the majority of restenosis after tracheal surgery are likely to occur within the first 4–6 postoperative weeks [40, 41]. We therefore believe that a median follow-up of 6 months was sufficient to detect most of the patients with restenosis after surgery. Another limitation is the small sample size that makes the analysis prone to errors through unknown confounding variables. Nevertheless, tracheal resections are rare in thoracic surgery and in terms of the disease, we can survey a relatively large number of patients.

## Conclusions

We analyzed a well-characterized cohort of patients that underwent airway surgery for TS. Perioperative morbidity was low without any mortality. The functional outcome was satisfactory. Prolonged ventilation was predictive for the development of complications. Primary idiopathic stenosis and female sex were risk factors for the development of recurrent stenosis.

## Data Availability

Raw data were generated at Thoraxklinik Heidelberg. Derived data supporting the findings of this study are available from the corresponding author [FE] on reasonable request.

## Abbreviations

RFS: Recurrence free-survival
TS: Tracheal stenosis
ITS: Idiopathic tracheal stenosis
